# Local Fields in Human Subthalamic Nucleus Track the Lead-up to Impulsive Choices

**DOI:** 10.3389/fnins.2017.00646

**Published:** 2017-11-23

**Authors:** John M. Pearson, Patrick T. Hickey, Shivanand P. Lad, Michael L. Platt, Dennis A. Turner

**Affiliations:** ^1^Center for Cognitive Neuroscience and Duke Institute for Brain Sciences, Duke University, Durham, NC, United States; ^2^Department of Neurology, School of Medicine, Duke University, Durham, NC, United States; ^3^Department of Neurosurgery, School of Medicine, Duke University, Durham, NC, United States; ^4^Department of Neurobiology, School of Medicine, Duke University, Durham, NC, United States

**Keywords:** STN, DBS, impulsivity, balloon analog risk task (BART), decision making, local field potential (LFP)

## Abstract

The ability to adaptively minimize not only motor but cognitive symptoms of neurological diseases, such as Parkinson's Disease (PD) and obsessive-compulsive disorder (OCD), is a primary goal of next-generation deep brain stimulation (DBS) devices. On the basis of studies demonstrating a link between beta-band synchronization and severity of motor symptoms in PD, the minimization of beta band activity has been proposed as a potential training target for closed-loop DBS. At present, no comparable signal is known for the impulsive side effects of PD, though multiple studies have implicated theta band activity within the subthalamic nucleus (STN), the site of DBS treatment, in processes of conflict monitoring and countermanding. Here, we address this challenge by recording from multiple independent channels within the STN in a self-paced decision task to test whether these signals carry information sufficient to predict stopping behavior on a trial-by-trial basis. As in previous studies, we found that local field potentials (LFPs) exhibited modulations preceding self-initiated movements, with power ramping across multiple frequencies during the deliberation period. In addition, signals showed phasic changes in power around the time of decision. However, a prospective model that attempted to use these signals to predict decision times showed effects of risk level did not improve with the addition of LFPs as regressors. These findings suggest information tracking the lead-up to impulsive choices is distributed across multiple frequency scales in STN, though current techniques may not possess sufficient signal-to-noise ratios to predict—and thus curb—impulsive behavior on a moment-to-moment basis.

## Introduction

The well-attested success of deep brain stimulation (DBS) in ameliorating the symptoms of neurological disorders, such as Parkinson's Disease (PD) and essential tremor has brought with it an intense interest in DBS as a treatment modality for other conditions, including obsessive-compulsive disorder (OCD) and treatment-resistant depression (Johansen-Berg et al., 2008; Malone et al., 2009; de Koning et al., 2011; Figee et al., 2013). This enthusiasm has also extended to an effort to identify biomarkers relevant to DBS targets for the cognitive symptoms of PD, in the hopes that alleviation of these symptoms could also be addressed by next-generation DBS devices (Berney et al., 2002; Hälbig et al., 2009; Abosch et al., 2011; Hoang et al., 2017). These impulsive dimensions of the disease are thought to arise from the interaction between cortical planning areas, such as pre-SMA/SMA and the subthalamic nucleus (STN), the site of DBS stimulation, via the so-called “hyperdirect” pathway (Nambu et al., 2002; Aron and Poldrack, 2006; Frank, 2006; Cavanagh et al., 2011; Zavala et al., 2014). Studies have shown that oscillations in the theta band (4–8 Hz) play a key role in mediating the influence of frontal cortical areas on decisions in the basal ganglia and likely represent cognitive, top-down aspects of the decision process (Frank et al., 2007; Ballanger et al., 2009; Cavanagh et al., 2011; Zavala et al., 2014, 2015). This complements an extensive literature on the role of beta band oscillations (13–30 Hz) in the pathophysiology of Parkinson's Disease, where it is known that such oscillations are suppressed by dopaminergic medication and effective DBS stimulation (Levy et al., 2002; Bronte-Stewart et al., 2009; Jenkinson and Brown, 2011; Whitmer et al., 2012). Similarly, power at these frequencies is reduced prior to voluntary movement, suggesting that such suppression is necessary for movement initiation (Jenkinson and Brown, 2011; Brittain et al., 2012).

However, while beta band activity has shown promising results as a biomarker suitable for training closed-loop DBS devices (Feng et al., 2007; Rosin et al., 2011; Santaniello et al., 2011), much less is known about the potential efficacy of stimulation targeting theta band. This stems, at least in part, from the more complex modulatory role posited for these oscillations in decision-making, where higher theta band power does not drive impulsivity but rather interacts with motivational signals entering the basal ganglia from the striatum and cortex (Cavanagh et al., 2011; Cavanagh and Frank, 2013; Zavala et al., 2014, 2015). Moreover, typical studies of these conflict-driven signals only report group averages, leaving open the question of variability across subjects and the robustness of these effects at the single subject level. Thus, the question of whether these signatures can be used and exploited at the level of individual subjects to predict and improve impulsive decisions in the face of conflict is a key one for the feasibility of next-generation DBS devices aiming to target and minimize cognitive side effects.

Here, we asked whether information about upcoming decisions might be disparately encoded by oscillations across the frequency spectrum by analyzing multi-channel intracranial recordings performed while patients with Parkinson's Disease underwent implantation of DBS. We asked patients to perform a version of the balloon analog risk task (BART) (Lejuez et al., 2002), which is often used to model impulsive choice behavior (Lauriola et al., 2013). In contrast to standard perceptual decision tasks (cf. also Zavala et al., 2014), decisions in our experiment took place over a time scale of several seconds, allowing us to track neural correlates of the deliberative process as they occurred preceding decisions. We report that both single units and LFP signals altered their activity over time during the decision period, with LFP power in multiple frequency bands ramping over the course of the final seconds preceding decision, with phasic changes following the response. We also found evidence at the group level for separate roles for theta band activity in regulating the decision process and beta band power in motor execution of the decision, consistent with prior studies. However, these patterns displayed wide variability at the group level, and a classifier trained to predict upcoming decision times failed to improve with the addition of field potential data. Together, these findings imply that decision-related information in the face of conflict is encoded at multiple frequencies within the basal ganglia, but development of additional, more reliable biomarkers may be needed as critical input for next-generation DBS devices designed to target impulsive decision-making.

## Materials and methods

We conducted intraoperative recordings in 15 patients undergoing implantation of DBS devices in the subthalamic nucleus for treatment of Parkinson's Disease. This study was carried out in accordance with the recommendations of the Duke University Medical Center Institutional Review Board with written informed consent from all subjects. All subjects gave written informed consent in accordance with the Declaration of Helsinki. The protocol was approved by the Duke University Medical Center Institutional Review Board. Patients were introduced to the study by a member of their treatment team. If interested, they were given further information by a study coordinator separate from the research team and afforded time to discuss with family prior to consent. Patients gave written consent preoperatively, either the morning of surgery or during a prior clinic visit. All patients who met the inclusion/exclusion criteria for DBS surgery were offered the opportunity to participate.

### Surgical procedures

All patients participating in the study (*N* = 15, 5 female, 10 male) underwent DBS implantation in the subthalamic nucleus (STN) for treatment of Parkinson's Disease (see Table 1 for details). Patients were off Parkinson's medication (i.e., sinemet, dopamine agonists) for >12 h preceding surgery and during the surgical procedure to enhance their symptoms as part of the routine clinical care.

**Table 1:** Patient characteristics.

**Age**	**Disease duration (years)**	**LEDD**	**Surgery side**
66–70	2.5	245	Unilateral, R
61–65	10	2,930	Bilateral
56–60	3	700	Bilateral
61–65	7	1,098	Bilateral
66–70	22	800	Bilateral
61–65	12	898.5	Bilateral
66–70	5	925	Bilateral
56–60	6	560	Unilateral, R
46–50	5	0	Unilateral, L
51–55	6	965	Bilateral
41–45	5	0	Unilateral, L^*^
66–70	14	510	Bilateral
56–60	9.5	1674.5	Bilateral
51–55	13	991	Bilateral
61–65	4	800	Bilateral

* *Previous R sided DBS*.

*M: male; F: female; LEDD: levodopa equivalent daily dose; R: right; L: left*.

Patients underwent detailed MRI scanning, typically followed by Leksell frame placement, with a high resolution CT scan used to localize the surgical target. A subset of patients underwent frameless implantation following placement of fiducial markers in the skull later referenced to MRI scans. In all cases, STN was identified by first mapping out indirect target coordinates (i.e., *X* = 11–12 mm off the midline, *Y* = 2 mm behind AC-PC midpoint, *Z* = 4 mm below AC-PC line) and then refining these initial coordinates by the area of decreased FLAIR density on sagittal and coronal reconstructions corresponding to STN, with small adjustments (typically 1–2 mm) in X and Y dimensions for best fit. Single-unit recordings were first performed to define the borders of the STN, according to standard electrophysiological criteria, with the goal of attaining at least 5.5–6 mm of typical STN multi-unit firing. Typically, 2–3 passes were performed. Localization was performed using single-channel tungsten microelectrodes (Frederick Haer, Brunswick, ME).

In most patients, data were collected during the process of STN localization. In some patients, data were collected from both sides of a bilateral DBS implantation (subjects 14, 16, 17). Once a well-isolated single unit was identified, the electrode would be left in place as the patient performed the behavioral task. Once data collection was complete, STN mapping would resume. In a subset of patients (*N* = 7; datasets 16.2, 17.2, 18.1, 20.1, 22.1, 23.1, 30.1), after full STN mapping, a 32-channel Pt/Ir microwire (35 μm diameter) array (Ad-Tech Medical Instruments, Racine, WI) was passed to the STN via an outer cannula (Patil et al., 2004) to a depth at which significant activity had been noted during initial localization. After allowing a few minutes for initial recordings to stabilize, the microwire array was slowly advanced through the cannula. Channels corresponding to putative single units were then selected and sorted, following which patients performed the behavioral task.

Following completion of the behavioral task, the DBS treatment electrodes were implanted. Patients undergoing bilateral DBS implantation were offered the opportunity to participate in the research twice, once for each implantation target. As a clinical routine, a brain computed tomography scan was performed within 12 h of the surgery procedure, and in no instance was a hemorrhage or other complication noted. Hence, the clinical risks of temporary placement of the 32 channel microwire array were demonstrated to be very low, as previously reported (Patil et al., 2004; Hanson et al., 2012).

### Behavioral task

We used a continuous time version of the BART (Lejuez et al., 2002), in which subjects must balance risk and reward in an attempt to maximize their score. In the original BART subjects choose whether to inflate a balloon, increasing its point value but also its risk of popping, or stop inflating and thereby add the current value of the balloon to their accumulated score. In the original task, each balloon had a maximal number of times it could be inflated (e.g., 10 or 20) and an equal chance of popping on each trial. If the balloon popped, a subject would receive no points for that balloon.

**Figure 1:**
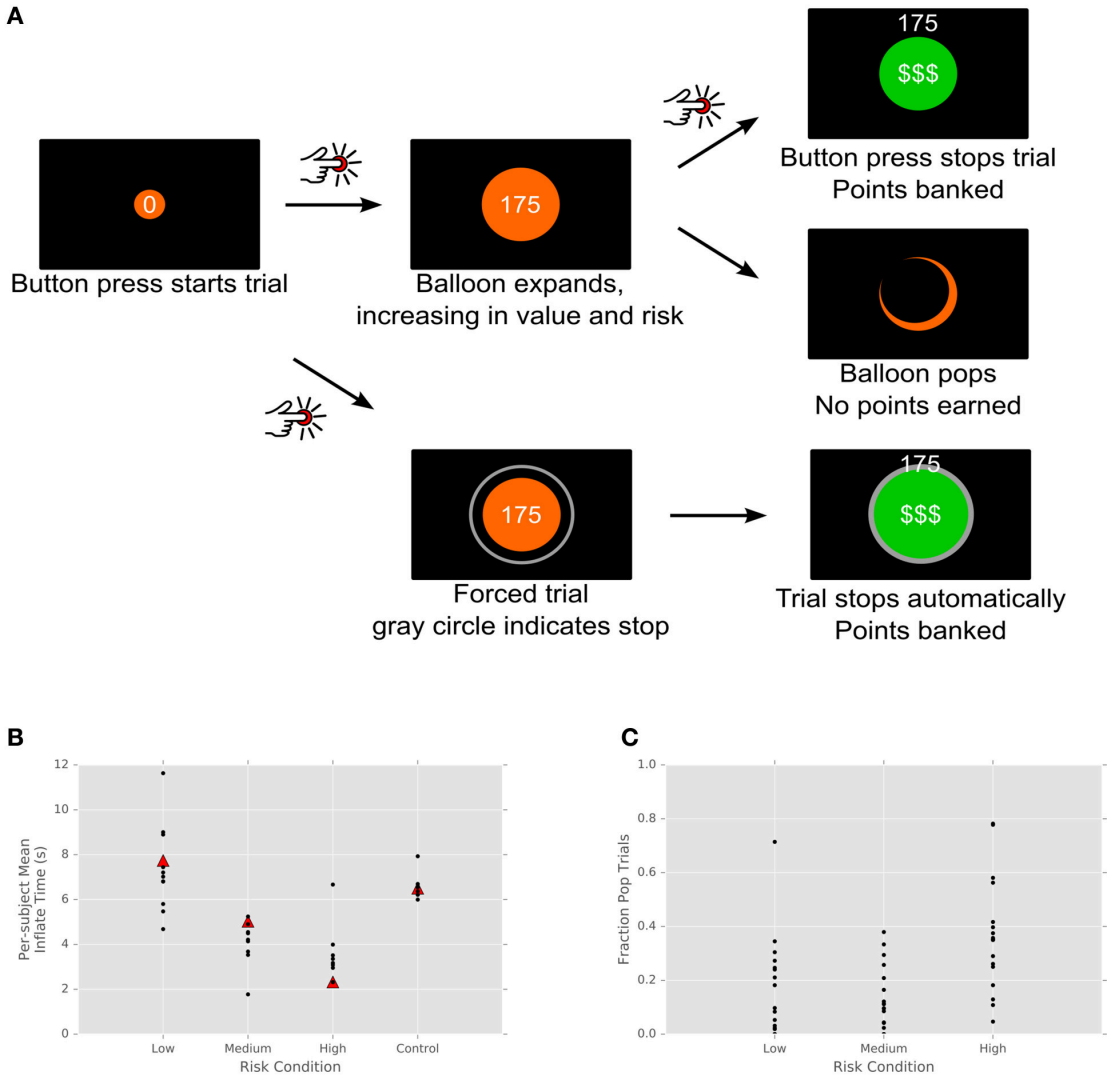
Task design and performance. **(A)** Participants pressed a button to begin inflating a virtual balloon onscreen. The value of the balloon grew with its size. Participants either clicked the joystick a second time, banking the balloon's value, or the balloon popped, earning no points. Balloons came in three colors, indicating different distributions of pop times and thus differing levels of risk. **(B)** Subject performance by risk condition. Each subject's mean inflate time is plotted for each risk condition and for control trials. Triangles indicate the mean of the actual pop time distribution, unknown to subjects. Control trials, in which the computer stopped the trial, show a tight cluster around the mean. **(C)** Fraction of pop trials for each subject. Each subject's fraction of popped balloons is plotted for each trial type.

Our variant modified this task to the constrained setting of the patient population in the operating theater. We designed a continuous-time version of the BART in which patients pressed a button to start inflating each balloon and pressed the button a second time to stop inflating and add its point value to their score (Figure 1A). In this version, balloons were divided into three risk levels, indicated by color. Each color corresponded to a distribution of maximal inflation (pop) times, with standard deviation proportional to the mean. This version proved much easier for patients to learn (pop times are more predictable) while preserving the cost/benefit tradeoff of the original task. Each balloon began with a value of 0 points, with its value increasing by 30 points/s. Pop times were drawn from a normal distribution on each trial (red: mean: 3 s, std: 0.9 s; orange: mean: 6.5 s, std: 1.95 s; yellow: mean: 10 s, std: 3 s). When patients successfully stopped the trial, the balloon turned green, a cash register sound played, and the balloon's point value was added to the accumulated score. If patients did not stop the trial in time, the balloon popped, resulting in a flash and a pop sound, and no points accrued.

For a subset of patients, two types of control conditions were added. In the first, 25% of balloons constituted forced trials. On these trials, a gray ring appeared at a fixed distance from the balloon, its radius drawn from the balloon's color-specific pop time distribution. That is, patients were explicitly shown a representative pop time of the balloon. On these trials, patients could not stop the balloon's inflation, and when the balloon's radius met the gray ring, the cash register sound played and patients received the current point value of the balloon. This subset of trials thus informed patients about the statistics of the task and attempted to control for motor anticipatory effects—since the outcome of the trial is certain and there is no need for stopping movement, motor planning should not play a role.

The second type of control trials involved gray (no-reward) balloons. Like the first type of control trial, these gray balloons were surrounded by a gray ring (with radius drawn from the intermediate/orange distribution). They functioned exactly as the first type of control trials, save that these balloons were worth no points. As a result, these trials control for effects of reward anticipation in the task.

Subjects were paid based on performance in the task, up to a maximum of $40. Subjects were informed in advance of the rank order of risk for each balloon color (yellow, orange, red), to minimize the need for within-task learning. Subjects were also given the opportunity to familiarize themselves with the task prior to the experimental session.

The task itself was coded in Matlab (The Mathworks, Natick, MA) using PsychoPhysics Toolbox (Brainard, 1997). These scripts are available online at https://github.com/jmxpearson/bart.

### Data acquisition and preprocessing

Data were recorded using a Plexon MAP system (Plexon, Inc., Dallas, TX) and the FHC Guideline 4000 (FHC, Inc., Bowdoin, ME). For single electrode recording, both high-pass and low-pass (local field potential; LFP) signal were recorded, with spikes extracted from the high-pass signal offline (see below). For multielectrode recording, LFP was recorded from all 32 channels and high-pass signal from the 16 most active channels, though all spike detection, and sorting was performed offline.

Prior to analysis, spike detection and sorting were performed via the WaveClus package (Quiroga et al., 2004) using a 4 standard deviation threshold based on robust (median-based) estimation of the noise. Putative units with <5 or >100 spikes/s average firing over the session were rejected based on established physiology of STN. Single units were identified based on reliability of spike waveforms (Tankus et al., 2009), with the remainder of units classified as multi-unit activity. Local field potentials (LFPs) were downsampled to 200 Hz. Recording artifacts were identified via either an aberrant increase in power or a sustained maximal amplitude (“railing”) and epochs containing these artifacts removed in subsequent analysis.

### Statistical analysis

#### Behavioral analysis

To calculate optimal behavior in the task, we made use of the fact that pop times in each condition were Gaussian distributed with known mean and variance, while rewards were proportional to elapsed time. That is, τ ~ *N*(μ, σ^2^) and *R* = α*t*, which implies an expected reward at time *t* proportional to both the elapsed time and the survival probability:

*E*[*R*|*t*] = α*t*(1− *CDF*(*t*; μ, σ^2^)) with CDF (*t*) the cumulative distribution function of the Gaussian with given mean and variance. For each risk level, we then numerically calculated *t*_*opt*_ by maximizing expected reward.

#### Spiking model

We modeled spike data as arising from a Generalized Linear Model with Poisson distribution and log link function:

$$\begin{array}{l} N\sim Poisson\left(f\Delta t\right)\\ \log\;f=\beta_{0}+\beta X+\gamma log\left(t\;-\;t_{0\;}+1\right) \end{array}$$

That is, counts in each Δ*t* = 50 ms bin were assumed to be Poisson distributed with rate *f*. Task variables were modeled as contributing linearly to the logarithm of *f*, such that the baseline firing rate of the cell is $f_{0}=e^{\beta_{0}}$ and the effect of adding a binary regressor *x*_*i*_ is a multiplicative gain $e^{\beta_{i}}$. As a result, our model predictors were time series, incorporating each bin for the entire experiment into a single regressor.

In addition to baseline spiking rate, our model includes an indicator term for the entire trial period (equivalent to the contrast between trial and inter-trial baseline), separate binary indicators for the inflation and outcome periods, a categorical regressor for outcome type (banked reward vs. no points), and a categorical regressor for trial type (risk levels high, medium, low, and control). Note that these regressors are not orthogonalized and suffer from multicollinearity, which we treat using regularization methods (see below). Finally, we include an effect of elapsed time within each trial, such that if *t* is the elapsed time and *t*_0_ the trial start time, the firing rate increases as $f=\alpha {\left(t\;-t_{0}+1\right)}^{\gamma }$, with α including the effects of all other variables.

We fit this model using elastic net regularization via the glmnet package in R (Friedman et al., 2010). In our results, we used 10-fold cross-validation to choose the values of λ and α. That is, we fit the model using 90% of the available data (time bins) and used the remaining 10% of data to assess its predictive performance. We then repeated this method 10 times using distinct random partitions of the data. For λ, we used the largest value (returned by glmnet) within one standard deviation of the value that produced the best performance, on average, for the cross-validation procedure. That is, we used the most parsimonious model (highest λ) statistically indistinguishable from the best-performing model. To determine α, we repeated the entire glmnet fitting 11 times, testing values of between 0.1 and 1 in steps of 0.1.

In addition, for comparison, we also calculated effect sizes for other regularization choices. These include the same model without regularization (more exactly, set to the minimum value used by the elastic net algorithm), with the value of lambda producing the best predictive performance, and the parsimonious model described above fit to shuffled data. In the last case, spikes counts for each time bin were randomly shuffled, removing any temporal correlations in the data that might be explained by our regressors.

#### Local field potentials

LFPs were preprocessed by first subtracting the mean across all channels at each time point from each channel to remove global artifacts, followed by bandpass filtering into typical frequency bands of interest as follows (frequencies in Hz): delta: (0.1, 4), theta: (4, 8), alpha: (8,13), beta: (13, 30), gamma: (30, 80). These signals were subsequently downsampled to 200 Hz and instantaneous power calculated via the square of the Hilbert transformed signal. Finally, time series for each channel were censored to remove microphonics, amplifier saturation, and other artifacts.

For peri-event LFP analyses, summary traces were computed by collecting samples around each event of interest (trial starts, trial stops, etc.), taking the median at each time point across events, and (for plotting purposes) standardizing the log power for each channel to have mean zero and unit variance. This last step allowed us to compare dynamics of the signal across channels despite variability in channels' dynamic range. Broadband signals were processed in the same manner, omitting the step of bandpass filtering. In some plots, traces were subsequently averaged across channels on the logged and z-scored scale. For instantaneous power analyses that collapsed across subjects, the group mean of subject means on the logged and *z*-scored scale was used. By normalizing prior to averaging, both of these choices give equal weight to each channel, rather than favoring channels with higher overall power. However, we also consider the case of average across channels without *z*-scoring in Supplementary Results.

Time-frequency plots were constructed via continuous wavelet transforms of the data. For analyses that collapsed across channels and subjects, individual channels were z-scored prior to averaging, so that channels with widely varying power contributed equally. We used a complex Morlet wavelet with *w* = 9 and logarithmically-spaced frequencies from 2.5 to 50 Hz. Power was calculated via the sum of squares of the real and imaginary parts of the wavelet transform and plotted on a decibel scale. To compensate for different levels of intrinsic power within frequency bands, the plotted signal was normalized by frequency band by the mean power present across all trials within that band in the interval B = (−1.5, −0.5), that is, from 1.5 s before to 0.5 s before the event of interest. Specifically, the plotted intensities are given by $I\;=\;10log_{10}\frac{P\left(f,t\right)}{P_{0}\left(f\right)}$ with *P*(*f, t*) = |*s*(*f, t*)|^2^, *s(f, t)* the wavelet-transformed signal, and *P*_0_*(f)* the mean power in the baseline interval. For time-frequency plots depicting the contrast between two conditions, no normalization was performed, so that the plotted intensities reflect $I\;=\;10log_{10}\frac{P_{1}\left(f,t\right)}{P_{2}\left(f,t\right)}$, the ratio of power between the two conditions in decibels.

For contrast time-frequency plots, we determined significant areas of relative activation via a cluster-based bootstrap algorithm (Nichols and Holmes, 2001). For each pixel, we calculated an F-statistic between the (decibel-scaled) power for the two conditions, using a 5 × 5 pixel local smoother to improve estimation of variance. We used *p*-value thresholds of 0.1 and 0.9 for these F-statistics to determine local clusters of activation. That is, pixels in the uppermost and lowermost deciles of cumulative probability density were selected for further analysis. Groups of contiguous pixels exceeding this threshold constituted a cluster, with cluster mass equal to the sum of log F-values for all pixels in the cluster. Distributions of this mass statistic under the null hypothesis were generated by randomly shuffling the labels of the two conditions and then repeating the entire analysis pipeline above (smoothing, F-statistic, thresholding, clustering). All clusters with mass statistic significant at the *p* < 0.05 threshold with respect to this null distribution were retained.

The LFP prediction algorithm made use of a modified sparse proportional hazards model. Inputs to the classifier were determined as follows: for each dataset, LFP in each channel was bandpass filtered into delta, theta, alpha, beta, and gamma bands as defined above, then decimated to 40 Hz, converted to instantaneous power via the Hilbert transform, censored for artifacts, log scaled, and standardized to 0 mean and unit variance. Regressors were the logged mean power for each (channel, band) combination for 0.5 s preceding each time point of interest. True positive events (coded as 1 s) were chosen as the response time for trials in which participants successfully stopped balloon inflation and received a reward. All other data points were non-events (coded as 0 s). We assumed these binary counts to be Poisson distributed within each time bin with instantaneous rate (at risk level *i*):

$$\begin{array}{l} \lambda_{i}\left(t\right)\;=\;h_{i}\left(t\right)\exp\left(\mu \;+\;X\left(t\right)\cdot \beta \right) \end{array}$$

with *h*_*i*_*(t)* the hazard function for the normal distribution with (fitted) parameters *m*_*i*_ *and s*_*i*_*, X(t)* the regressors (LFP power in each band, channel) at time *t*, and μ and β model parameters distributed according to a horseshoe prior (Carvalho et al., 2009). The utility of the horseshoe prior lies in its privileging of sparse solutions for the regression weights, allowing us to simultaneously perform inference and model selection. That is, channels and frequency bands are parsimoniously shrunk to 0, as in the LASSO model above. Note in particular that the use of instantaneous log power for *X(t)* implies that the event rate is the equal to the hazard rate modulated by a weighted geometric mean of momentary power.

We fit these models using the Stan probabilistic programming language (Carpenter et al., 2016) using four chains of 2,000 iterations each with a burn-in period of 1,000 samples (half the total) and a thinning fraction of five. Convergence was assessed by means of effective sample sizes and the Gelman-Rubin statistic (Gelman and Rubin, 1992).

## Results

As expected, subjects balanced risk and reward, with longer inflation times for less risky balloons. In addition, nearly all subjects exhibited risk aversion across each of the categories, with mean trial times below optimal for the low and medium risk conditions (but above in the high risk condition; Figure 1B). This is consistent with behavior observed in the standard BART (Lejuez et al., 2002; Lauriola et al., 2013), where the level of risk aversion is the measure of interest, since it has been shown to correlate inversely with real-world measures of risk-taking. This is also consistent with Figure 1C, which shows the fraction of trials for each subject that resulted in no points (i.e., popped balloons). In the standard BART, where the total number of trials is fixed, optimal strategy dictates that subjects should inflate balloons to half their maximal size—that is, on average, half of trials should result in pops. In our modified paradigm, by contrast, the number of trials was time-limited, with the result that subjects were incentivized to adopt a strategy of shorter, more numerous trials with higher probability of reward. However, what is most important for our subsequent analysis of neural data is not whether subjects pursued behaviorally optimal strategies but the fact that varying risk levels in the task resulted in a range of decision times, varying (roughly) between 1 and 12 s. Most importantly, these responses were self-initiated. Rather than rapid responses to external cues, they were the result of deliberative behavior, permitting us to examine the time evolution of signals underlying the process of decision formation within STN.

**Figure 2:**
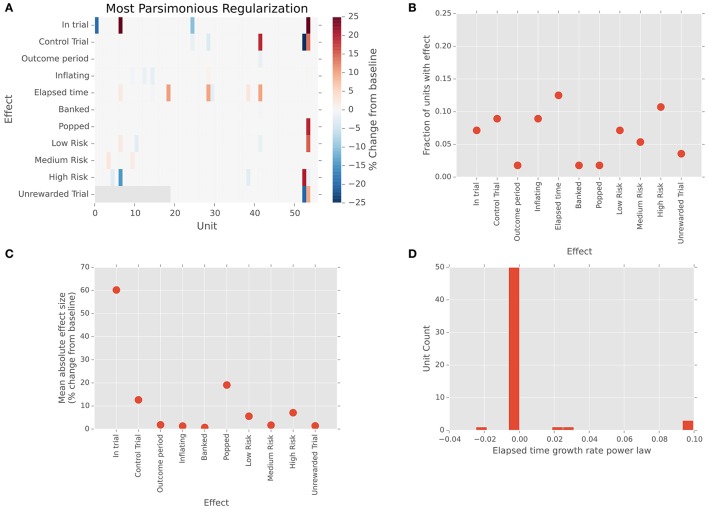
Single units in STN show sparse responses to task variables. **(A)** Effect sizes for each unit and task variable in the firing rate model. Neutral colored tiles indicate no effect. Colors indicate percent change in firing relative to inter-trial baseline. **(B)** Fraction of units exhibiting each effect. **(C)** Mean absolute effect size (percent change from baseline) across units in which that effect was present in the model. **(D)** Change in firing rate over time. Histogram of firing rate effect, expressed as percent change relative to baseline. Effects are calculated for a representative elapsed time of 5 s, based on the fitted exponent gamma in the power-law growth model $f=\alpha {\left(t\;-t_{0}+1\right)}^{\gamma }$.

We recorded 56 (*N* = 48 single, 8 multi-) units from our patient population. All but one of these units was determined to lie within STN boundaries based on results of the intraoperative mapping process and pre-surgical MRI-based targeting. (One unit, with regular baseline firing rate of 150 spikes/s and an absence of bursting, was most likely sampled from the nearby substantia nigra pars reticulata.) We analyzed spiking activity of the STN units via a regularized, cross-validated GLM approach that incorporated all task variables into a single model. Figure 2A shows the effect sizes for each unit and task variable, color-coded as a percent change from baseline firing. Individual cells show differences in firing between risk levels, as well as between reward and no-reward trials and between standard (free choice) and control (no reward) trials. That is, local firing rates are sensitive to risk and the reward, as well as to the difference between trials on which a motor action must be made and those in which passive viewing alone is required. Clearly, the effects are sparse throughout the population, as is typical for neurons in basal ganglia. However, it is also important to note that the regularization and cross-validation approach we have used is extremely conservative; a similar approach using the best-performing (not most parsimonious) regularization found similar effects much more strongly represented, with roughly 80% of cells exhibiting each effect. Along the same lines, a shuffle-corrected version of the same analysis produced zero false positive responses (Figure S1). That is, multiple checks indicate that the sparse effect matrix above represents robust effects in our data, not statistical artifacts of our analysis.

We found neurons responsive to a variety of task-related variables, including those active for the difference between task and baseline (*N* = 4/56, 7%) and in the contrast between risk levels (Figure 2B; *N* = 11/56, 20%). We likewise found a range of effect strengths, with the effect of task vs. baseline and popped balloons largest (Figure 2C). Most intriguingly, the most commonly represented effect in our neural sample was the effect of elapsed time on neural firing. Roughly 14% (*N* = 8/56) of cells in our population showed small to moderate changes in firing rate over the course of balloon inflation, with effects on the order of a few percent to 20% changes in gain for a representative inflation time of 5 s (Figure 2D). These findings are consistent with previous reports of increased oscillatory power in STN in tasks requiring rapid responses (Cavanagh et al., 2011; Frank et al., 2015), but to our knowledge, this is the first single unit evidence of this mechanism operating over longer decision timescales in humans.

Intrigued by these findings in single units, we looked for further evidence of ramping activity at the population level preceding decisions. LFP are thought to measure voltages due to the summed activity of neurons in a local region around the electrode tip (potentially including some long-range effects) (Kajikawa and Schroeder, 2011). As such, they offer a glimpse into the rate and magnitude of local neural processing in a region. We recorded LFPs simultaneously with our single units, in some cases from up to 32 channels simultaneously. We asked whether these signals contained information about upcoming stop decisions, and whether patterns of local activity might serve to predict these decisions in a data-driven way.

**Figure 3:**
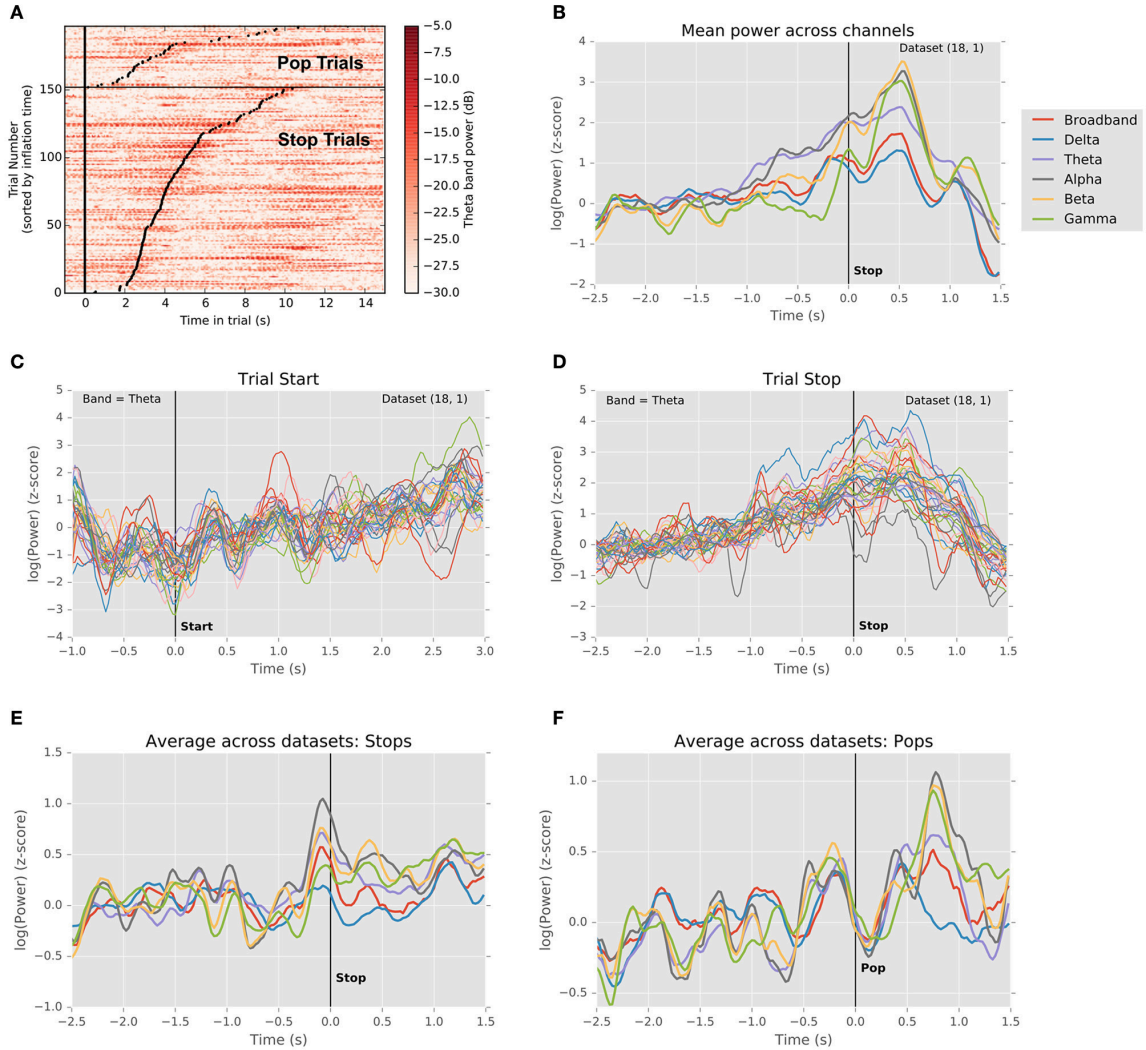
Changes in local field potential over the course of the trial. **(A)** Raster plot of power in the theta frequency range (4–8 Hz) for each trial in a single dataset (18.1). Each line represents one trial, with trials ordered vertically by duration. Successful stops are plotted below unsuccessful stops (balloon pops). Left vertical black line indicates trial start. Black dots indicate trial stops. Color indicates theta-band power in dB. A clear increase in theta power precedes successful stops. **(B)** Normalized LFP power rises preceding stops. Traces depict mean (across channels) of medians (across successful stop trials) LFP power in delta, theta, alpha, beta, and gamma frequency bands, as well as broadband signal, aligned to the time of the stop (*t* = 0). Power in theta band rises preceding the stop, falling gradually thereafter, whereas power in alpha and beta bands dips prior to the stop, rises sharply thereafter, and falls abruptly during the outcome period. **(C)** Normalized theta band LFP power in each channel aligned to trial start (t = 0). Traces indicate medians across trials (one trace per channel). Theta power dips just prior to trial onset and begins to rise as the balloon inflates. **(D)** Normalized theta band power in each channel aligned to trial stop. Traces represent medians across successful stop trials (one trace per channel). Power increases until the stop, following which it declines toward its pre-trial baseline. **(E)** Grand mean (averaged across datasets) trajectory of power in different frequency bands preceding voluntary stops (*t* = 0). Conventions are as in **(A)**. Note that multiple frequency bands exhibit changes in power preceding stops. **(F)** Grand mean (averaged across datasets) trajectory of power in different frequency bands preceding involuntary stops (i.e., pops; *t* = 0). Conventions are as in **(A)**. Note the sharp rise in power during the outcome phase, similar to the rise in power preceding voluntary stops.

Figure 3 explores this question by depicting LFP power in the theta frequency band (4–8 Hz) as a function of time on each trial for a single subject. Theta band activity measured by scalp EEG has been shown to be correlated with reaction times in a learned stimulus association task (Cavanagh et al., 2011; Zavala et al., 2013, 2014), where it was hypothesized that this is due to the influence of mediofrontal cortex on integration of information in STN via the hyperdirect pathway. Here, in a self-paced decision context, we see a similar pattern recapitulated in theta-band power within the STN. Each line of the image in Figure 3A represents a single trial. The vertical line marks the start of each trial, while each black dot marks its end. Trials have been grouped by outcome, and ordered by duration. For successful stops, theta band power clearly increases preceding stop movements, with some activation lasting into the outcome period. By contrast, this pattern is largely absent prior to unexpected stops (popped balloons). Like the data from single units, this suggests that local activity in STN begins to rise during the decision interval, peaking around the time of movement.

Figure 3B extends this analysis to other frequency bands, depicting the trial- and channel-averaged power surrounding trial starts and successful stops for the same subject as in Figure 3A. Clearly, power ramps over the 2 s preceding stops across frequency bands (*N* = 7/7 subjects, *p* < 0.05, *t*-test for regression slope), with alpha and theta power exhibiting a sustained rise. Intriguingly, for this subject, beta and gamma power begin their rise relatively late, <500 ms prior to the stop and coincident with a local peak in delta power. This is consistent with the idea that, for instance, beta band power increases precede motor movement (Jenkinson and Brown, 2011; Brittain et al., 2012) and that theta band power is related to a threshold for evidence accumulation (Cavanagh et al., 2011; Frank et al., 2015), though clearly peri-event activity is not confined to only a single frequency band. In Figures 3C,D, for the specific case of theta band, we see that this rise in power begins well before, at trial onset, and that a dip in power precedes the initiation of the trial. As a result, rises in theta begin well before the time of movement, suggesting that this process pertains to planning, not simply movement initiation. Likewise, at the population level, ramping activity preceded stops, and sharps increases in power followed pops, consistent with previous findings (Figures 3E,F; Cavanagh et al., 2011; Tan et al., 2014; Zavala et al., 2014, 2015).

**Figure 4:**
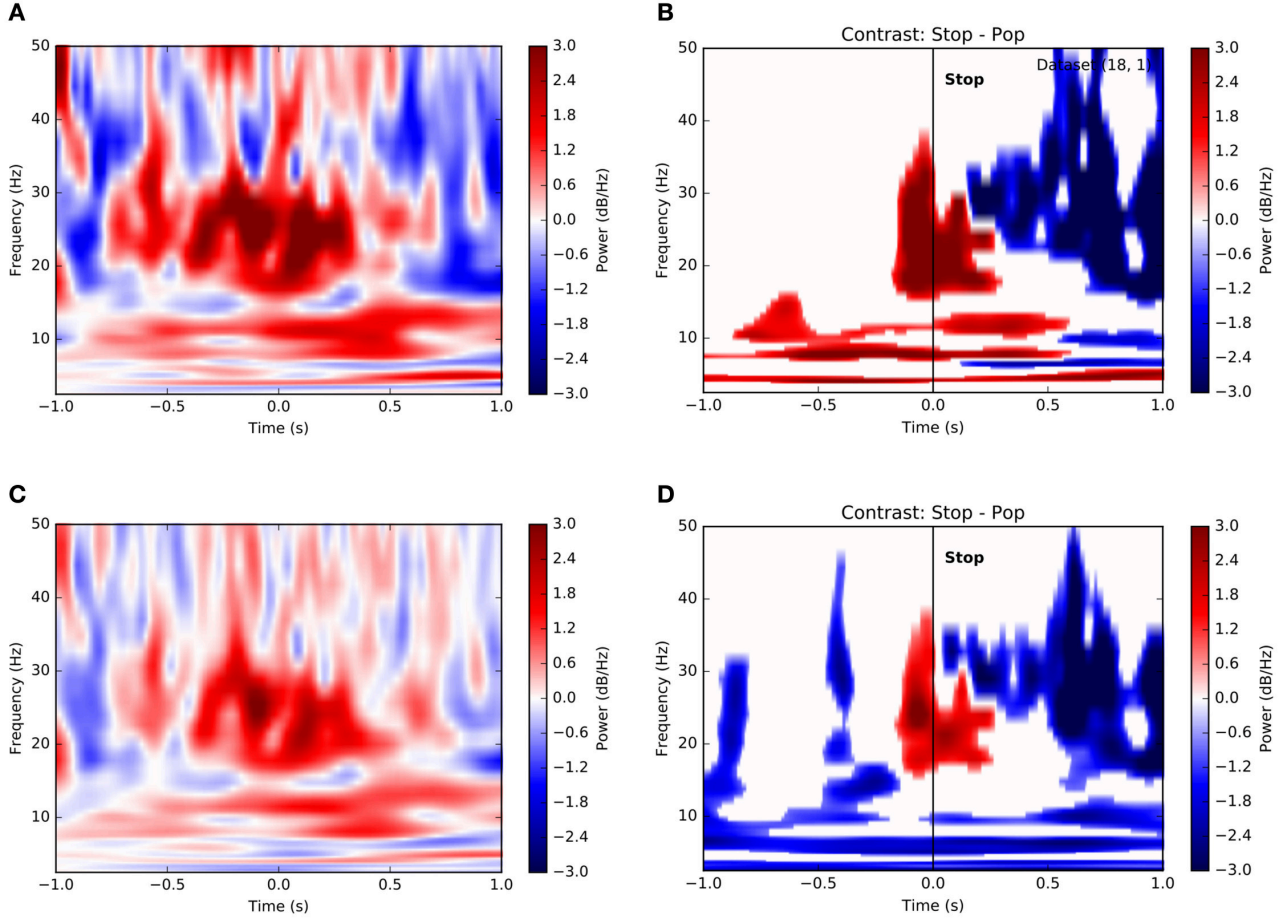
Time-frequency contrasts show dissociable roles for different frequency bands in stopping. **(A)** Time-frequency plot of power on successful stop trials aligned to time of stop (*t* = 0) for a single subject. Color depicts change normalized change in power relative to pre-trial baseline in decibels (see Methods). Increases in both low-frequency power and beta band activity precede the stop. **(B)** Significance-thresholded time-frequency contrast plot depicting differences in power between successful and unsuccessful stop (pop) trials for a single subject. Conventions and subject are as in **(A)**. Clusters represent statistically significant changes in activity between conditions (see Methods). Here again, voluntary stop decisions are marked by early increases in low-frequency power and peri-stop increases in beta band power more proximal to movement. **(C)** Equivalent of **(A)** using all trials across all subjects. **(D)** Equivalent of **(B)** using all trials across all subjects.

Observing that population averages in Figures 3E,F exhibited ramping activity across multiple frequencies, we asked whether such an integrative signal corresponded specifically to theta-band activity or whether it might involve oscillations at multiple scales. Figure 4A is a time-frequency plot showing channel- and trial-averaged LFP power preceding successful stops for a single subject. Clearly, power increases both at frequencies below 15 Hz and within the beta band in the second preceding decision, peaking just after decision at the time of reward. Indeed, this pattern remains when we consider the contrast between successful stops and unanticipated stops (popped balloons) (Figure 4B). Again, significant increases in low-frequency (<15 Hz) and beta band power distinguish between the two conditions, suggesting that decisions to stop require an increase in theta- and alpha-band activity along with a concomitant increase in beta power, as has been reported elsewhere (Bronte-Stewart et al., 2009; Jenkinson and Brown, 2011; Whitmer et al., 2012; Bastin et al., 2014). In fact, very similar results hold for a population-level analysis based on the average across all trials and all subjects (Figures 4C,D).

It may be considered surprising that Figure 4 depicts an increase in beta-band power, as opposed to the decrease reported in, e.g., Cavanagh et al. (2011) and elsewhere. In fact, this results from our choice of normalization. In Figure 4, we have first normalized the variance within each recording channel prior to averaging, which treats each signal on an equal footing. However, a raw average across, without normalization, shows a decrease in beta around the time of stops (Figure S3). This suggests that the largest modulations of LFP power in STN are negative, though weaker positive modulations may be widespread.

There is also suggestive evidence for this interpretation from a subset of participants who performed control conditions with both voluntary and forced stops. Figure S2A shows that in this case, voluntary stops, which involve both decision and motor movement on the part of participants, exhibit greater power in the beta and theta bands preceding stops. That is, the LFP effects noted above are related to the decision and movement process, not simply the temporal structure of the trial. Similarly, when comparing two identical motor actions, the movements at trial start and trial stop (Figure S2B) the differences in power between the two conditions appear to be quite similar to the overall activity for end-of-trial stops, suggesting that these effects pertain to the decision process itself. Thus, converging evidence supports the notion that gradual increases in low-frequency power are reflective of the deliberative decision process, while beta power changes precede movement initiation, followed by a rapid rise in beta post-movement and a fall across all bands after reward.

**Figure 5:**
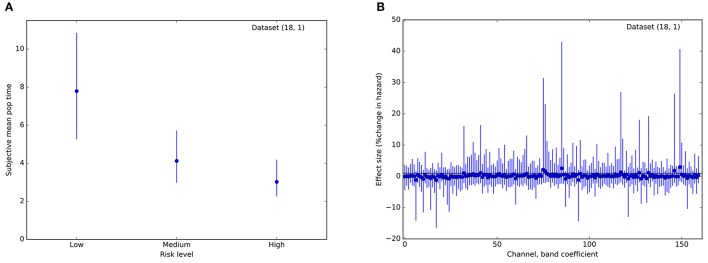
Results from a Bayesian proportional hazards regression model. **(A)** Risk level (balloon color) modulates hazard rate. Plot of estimated mean parameter for a hazard function based on a normal distribution of event (stop) times. Dots indicate median of the posterior distribution, lines indicate 95% posterior credible intervals. **(B)** Posterior estimates of regression coefficients (one per frequency band, channel pair) for the model. Effect sizes are measured in percent change in hazard rate per standard deviation of the regressor (regressors were standardized to have variance 1). Conventions are as in **(A)**. For all coefficients, the 95% credible interval includes 0, indicating that local field power did not improve predictions of stop time. These plots are for a single representative dataset (18, 1).

Therefore, we asked whether activity across multiple channels might collectively be harnessed to predict trial stops at the single-subject level. Using a survival analysis approach, we modeled the time to event (voluntary stop) as dependent upon an increasing hazard rate. Trials on which the balloon popped were treated as censored data, with the voluntary stop time unobserved. Most importantly, we assumed that the instantaneous probability of stopping was modulated by a weighted geometric mean of momentary power within each frequency band and channel. That is, the probability of a stop increased with time in a manner specific to each balloon color, modulated by a weighted combination of local neural activity. As depicted in Figure 5A, models for most subjects exhibited different hazard rates for the different risk levels, resulting in earlier stops for riskier balloons. However, with only a single exception, the coefficients encoding neural effects on stopping were not appreciably different from 0 for any subject, frequency band, or channel (95% credible interval for β overlapping 0; e.g., Figure 5B). Thus, while STN activity appears to track the deliberative process by ramping power across multiple frequency bands, this activity does not appear to be specifically predictive of the stop decisions themselves on a single-trial basis.

## Discussion

By recording directly from humans undergoing invasive brain surgery, we have demonstrated not only the presence of single units within the basal ganglia that increase firing over the course of the decision process, but that population measures like the local field potential also exhibit ramping activity during the process of deliberation. This finding is consistent with previous experimental (Frank et al., 2007, 2015; Cavanagh et al., 2011; Brittain et al., 2012; Zavala et al., 2013, 2014) and modeling (Frank, 2006) work positing a role for the mediofrontal-hyperdirect pathway in stopping behavior, but extends these findings beyond the realm of cued reaction time tasks to the longer timescale of deliberative decisions, confirming the applicability of experimental and modeling predictions to human cognitive tasks.

We likewise found single units and LFP signals responsive at the time of reward or negative outcomes, with stronger effects for balloon pops than rewards. This is consistent with single unit studies in animals showing responses to both rewards and error trials (Isoda and Hikosaka, 2008; Lardeux et al., 2009; Baunez and Lardeux, 2011), and likely reflects widespread neural activity in response to reinforcing outcomes across the brain. However, the most prevalent single unit response was to elapsed time in the trial, suggesting that STN not only plays a role in stopping behavior, but also encodes critical information related to upcoming choice during more prolonged deliberative processes.

Likewise, much debate has focused on the role and relative importance of oscillatory activity within STN and the basal ganglia more broadly. Activity within the beta band is known to be related both to upcoming motor movement, with desynchronization preceding movement onset (Jenkinson and Brown, 2011), and to the particular pathophysiology of Parkinson's Disease, where beta activity is known to be enhanced, while medications and effective DBS can suppress beta activity with symptomatic treatment (Bronte-Stewart et al., 2009; Jenkinson and Brown, 2011; Whitmer et al., 2012). In addition, theta band activity recorded in scalp EEG, thought to originate over medial frontal cortex, perhaps in pre-SMA/SMA, has been linked to cortical control over stopping in the basal ganglia (Aron and Poldrack, 2006; Cavanagh et al., 2011; Zavala et al., 2013, 2014), consistent with single-unit studies in non-human primates (Isoda and Hikosaka, 2007, 2008). Previous work in a cued response task showed both theta enhancement and beta suppression prior to responses, with theta linked via computational modeling to decision threshold in a drift diffusion model (Cavanagh et al., 2011; Zavala et al., 2014; Frank et al., 2015). Our findings complement this work by demonstrating a gradual increase in LFP power across multiple frequency bands preceding self-paced decisions, reflecting widespread STN activity. Thus, while beta desynchronization does occur before motor movement, antecedents of the decision output occur within multiple frequency bands after the decision process is activated. Furthermore, we provide additional evidence for a posited dissociation between beta/motor and theta/cognitive inputs to STN, consistent with the hypothesis that these signals reflect decision-related information from cortical afferents, likely in primary, associative motor, and midline limbic areas.

Several previous studies have delineated a specific role for theta-band activity in STN as setting a threshold for stopping behavior in the context of the drift-diffusion model (Frank, 2006; Cavanagh et al., 2011; Zavala et al., 2013). Evidence suggest that this activity represents top-down inputs from medial prefrontal cortex, with stronger theta activity present in the case of increased conflict. However, in our data, theta activity clearly increases over the course of the trial, favoring a role for theta in tracking the decision process rather than setting a threshold. Several observations may explain this discrepancy. First, theta may indeed play a dual role in both the accumulation process and threshold-setting, one which our design did not have sufficient power to detect. Second, the distinct demands of our task, which required neither a speeded response nor a forced choice, might have resulted in qualitatively different patterns of network activity. Likewise, our task offered only a single response option, asking participants a question of “when” rather than “which,” and so likely failed to recruit mechanisms responsible for mediating response conflict. Finally, another study of response conflict in STN on somewhat longer timescales (Zavala et al., 2014) found no correlation between theta band activity and response time, consistent with our results. However, we are agnostic as to whether these early onset signals represent conflict or some other psychological construct, since many similar ideas like dread, risk, or reward anticipation may plausibly rise during the course of our task, and none constitutes a mechanistic explanation. Future studies will no doubt be needed to elucidate the specific function or functions of theta-band activity in STN.

However, there are several limits to the current study. First, as noted above, there remains some uncertainty as to the exact role played by frontal theta in conjunction with the STN. While the focus has often been on conflict and stopping, Brittain and Brown (2014) have also suggested a relationship with attention. Huebl et al. (2011) found a link between alpha band activity and emotional state. Indeed, the signals we have identified as part of the deliberative phase span frequency bands and are no doubt involved in multiple processes, many of which are difficult, if not impossible to dissociate in a single study. Finally, the positions of our microwire electrodes are distributed at random throughout STN but may not sufficiently span the more medial areas associated with limbic and motivational inputs that we expect to contribute to impulsive decisions. Nonetheless, what is important for our purposes is that our task successfully elicits activity typically observed in studies designed to study impulsive decisions and so we are in a position to ask whether such signals are capable of predicting behavior at a single-trial level.

Most importantly, our results underscore the need for identifying additional high-quality biomarkers for closed-loop DBS systems aiming to treat impulsive side effects of PD. A number of closed-loop systems have focused on suppression of beta-band activity as a marker for motor pathophysiology, but an endogenous electrophysiological surrogate marker for impulsivity remains elusive (Jenkinson and Brown, 2011; Rosin et al., 2011). Even with a much higher channel count than possible with typical DBS devices, we were unable to effectively predict stopping (that is, to improve our probabilistic prediction of the stop time) on a single trial basis by incorporating neural signals from all channels and all frequency bands. Given the multiply attested relationship between beta band activity and stopping, this is perhaps surprising, but can perhaps be explained by two observations: First, our experiment used high impedance microelectrode wires as opposed to larger DBS contacts, resulting in less averaging and more spatially local signals. It is possible that this coarse-graining may be helpful in mitigating noise and deriving more predictive biomarkers in other DBS studies. Second, most studies report signals averaged over a larger number of trials. While reliable, such signals may be dwarfed by noise on an individual trial basis and thus of little utility for prediction. This single trial limitation could be overcome by averaging across several trials for improved predictions, which may still be feasible to implement in a slower cognitive feedback system. Finally, more sophisticated algorithms, equipped with larger training data sets like those being collected from already implanted DBS devices, may be able to extract more reliable signals from numerous cross-channel correlations. Clearly, further work is needed to identify such signals for the next generation of closed-loop DBS devices.

## Author contributions

JP, PH, SL, MP, and DT designed research. JP, PH, SL, and DT collected data. JP analyzed data. JP, PH, SL, MP, and DT wrote the manuscript.

### Conflict of interest statement

The authors declare that the research was conducted in the absence of any commercial or financial relationships that could be construed as a potential conflict of interest.
