# Supplementary material for: Local Fields in Human Subthalamic Nucleus Track the Lead-up to Impulsive Choices
Source: Front Neurosci. 2017 Nov 23;11:646. doi: 10.3389/fnins.2017.00646 (PMC5703842; doi:10.3389/fnins.2017.00646)
Supplement: Supplementary file 1 [file DataSheet1.PDF]

Supplementary Material

Local fields in human subthalamic nucleus track the lead-up to impulsive choices

John M. Pearson*, Patrick T. Hickey, Shivanand P. Lad, Michael L. Platt[1,4], and Dennis A. Turner

*** Correspondence:** Corresponding Author: john.pearson@duke.edu

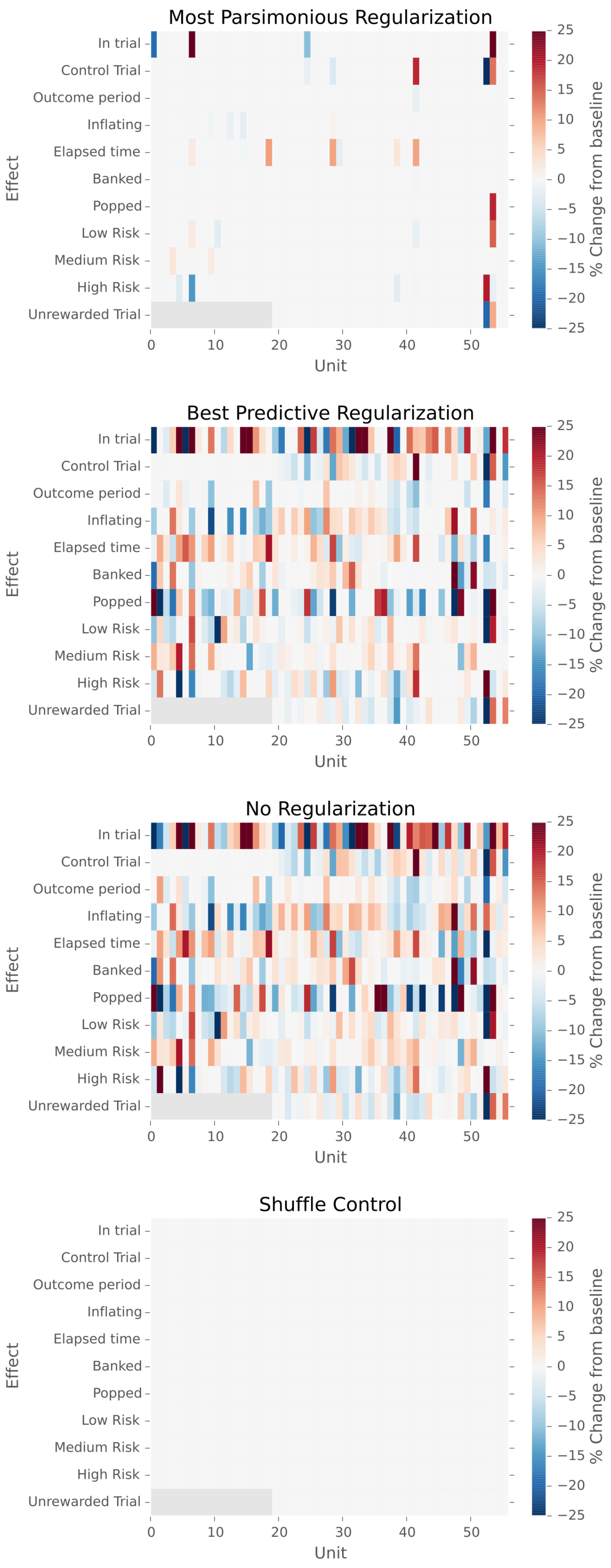
Most Parsimonious Regularization
Best Predictive Regularization
No Regularization
Shuffle Control
In trial
Control Trial
Outcome period
Inflating
Elapsed time
Banked
Popped
Low Risk
Medium Risk
High Risk
Unrewarded Trial
Effect
Unit
0
10
20
30
40
50
% Change from baseline
25
20
15
10
5
0
−5
−10
−15
−20
−25

Supplementary Figure 1. Comparison of regularization methods for effects of task variables on spiking. Conventions are as in Figure 2A. Depicted are effect sizes for the most parsimonious regularization (i.e., Figure 2A), the regularization with best predictive power on the holdout data, the model with no regularization (a generalized linear model), and a regularized model applied to shuffled data in which the relationship between time bins and regressors has been randomized (see Methods).

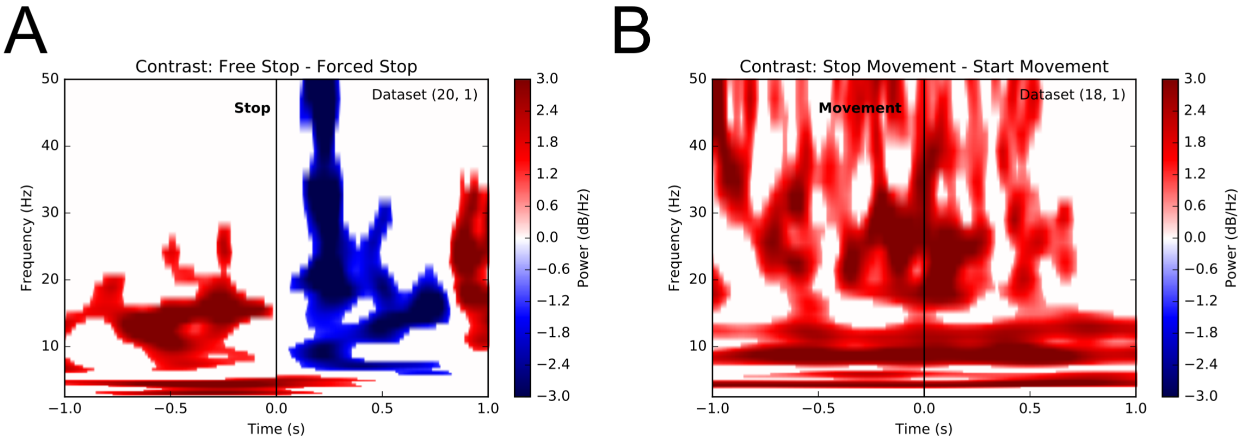

Supplementary Figure 2. (A) Time-frequency contrast plot depicting differences between voluntary stops and control trials (forced stops), for which no motor movement was made. Conventions are as in Figure 4. A prominent increase in beta-band power prior to movement onset similar to that in Figure 3B is visible around the time of stopping. **(B)** Time-frequency contrast plot comparing similar motor movements made to stop and start the trial (t = 0). The pattern is similar to that in Figure 4A, C, suggesting that the activity in those plots is largely attributable to the decision and not pure motor execution.

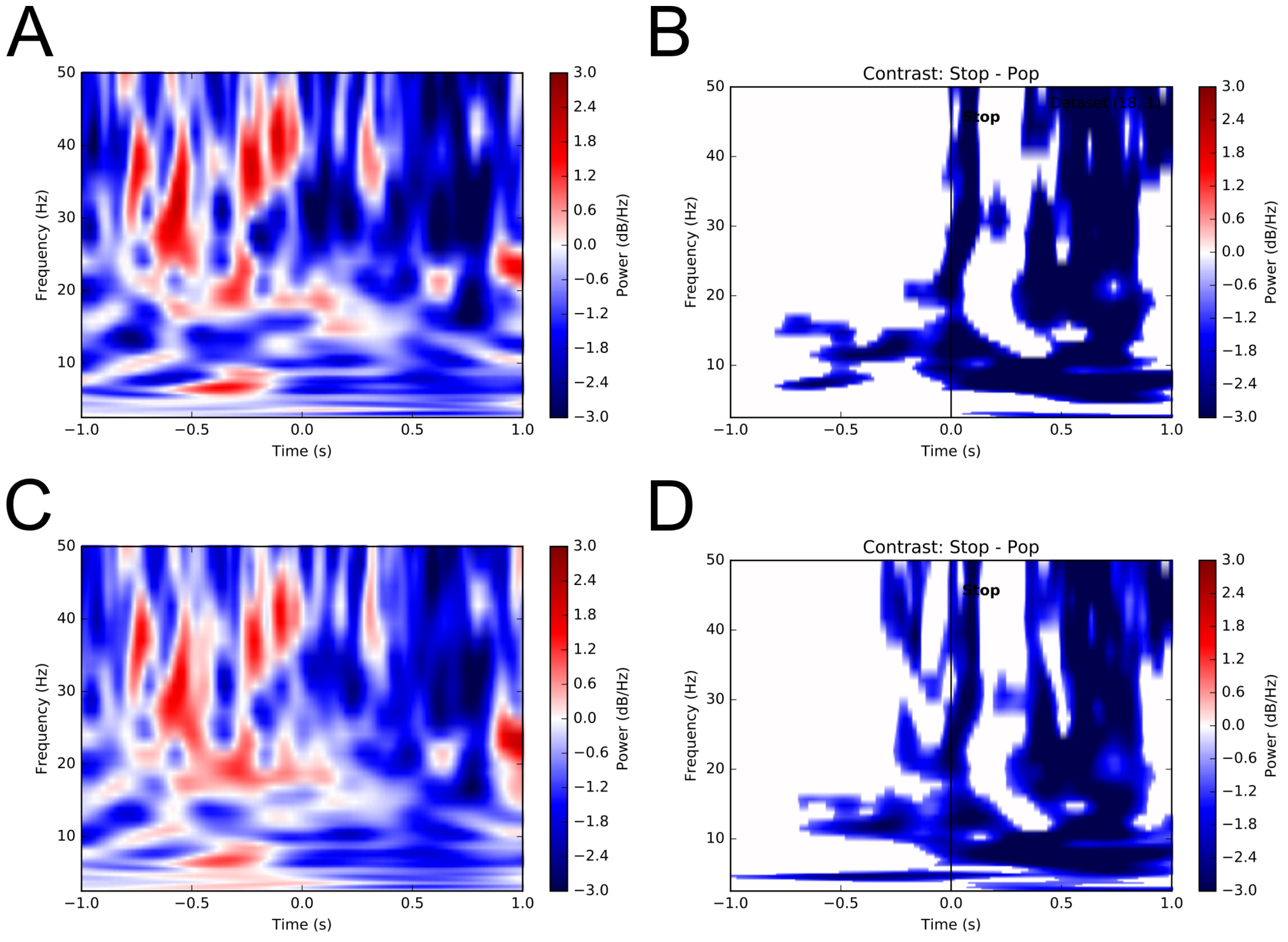

Supplementary Figure S3. Time-frequency contrasts show dissociable roles for different frequency bands in stopping. Same as Figure 4, but channels are not variance-normalized prior to averaging. Here, we see that the overall effect is one of decrease in beta-band power prior to stops, as reported elsewhere. Conventions are the same as in Figure 4 of the main text.
